# Supplementary material for: Different factors control long-term versus short-term outcomes for bacterial colonisation of a urinary catheter
Source: Nat Commun. 2025 Apr 26;16:3940. doi: 10.1038/s41467-025-59161-y (PMC12033313; doi:10.1038/s41467-025-59161-y)
Supplement: Supplementary file 2 — Reporting summary [file 41467_2025_59161_MOESM2_ESM.pdf]

nature portfolio

Corresponding author(s): Rosalind J Allen

Last updated by author(s): 2025/03/11

Reporting Summary

Nature Portfolio wishes to improve the reproducibility of the work that we publish. This form provides structure for consistency and transparency in reporting. For further information on Nature Portfolio policies, see our Editorial Policies and the Editorial Policy Checklist.

Statistics

For all statistical analyses, confirm that the following items are present in the figure legend, table legend, main text, or Methods section.

n/a	Confirmed	
☐	☒	The exact sample size (*n*) for each experimental group/condition, given as a discrete number and unit of measurement
☐	☒	A statement on whether measurements were taken from distinct samples or whether the same sample was measured repeatedly
☒	☐	The statistical test(s) used AND whether they are one- or two-sided<br>*Only common tests should be described solely by name; describe more complex techniques in the Methods section.*
☒	☐	A description of all covariates tested
☒	☐	A description of any assumptions or corrections, such as tests of normality and adjustment for multiple comparisons
☐	☒	A full description of the statistical parameters including central tendency (e.g. means) or other basic estimates (e.g. regression coefficient) AND variation (e.g. standard deviation) or associated estimates of uncertainty (e.g. confidence intervals)
☒	☐	For null hypothesis testing, the test statistic (e.g. *F*, *t*, *r*) with confidence intervals, effect sizes, degrees of freedom and *P* value noted<br>*Give P values as exact values whenever suitable.*
☒	☐	For Bayesian analysis, information on the choice of priors and Markov chain Monte Carlo settings
☒	☐	For hierarchical and complex designs, identification of the appropriate level for tests and full reporting of outcomes
☒	☐	Estimates of effect sizes (e.g. Cohen's *d*, Pearson's *r*), indicating how they were calculated

Our web collection on statistics for biologists contains articles on many of the points above.

Software and code

Policy information about availability of computer code

Data collection	Data was generated by custom code developed within the study, available in the Zenodo repository with the identifier doi.org/10.5281/zenodo.15001619
Data analysis	Data was analysed in Python 3.11.9, using the libraries: numpy 1.26.4, pandas 2.2.2, matplotlib 3.8.4, seaborn 0.13.2, SALib 1.5.1. Code used for the data and sensitivity analysis is available in the Zenodo repository with the identifier doi.org/10.5281/zenodo.15001619 . Statistical analysis was performed in Stata 18.5.

For manuscripts utilizing custom algorithms or software that are central to the research but not yet described in published literature, software must be made available to editors and reviewers. We strongly encourage code deposition in a community repository (e.g. GitHub). See the Nature Portfolio guidelines for submitting code & software for further information.

Data

Policy information about availability of data

All manuscripts must include a data availability statement. This statement should provide the following information, where applicable:

- Accession codes, unique identifiers, or web links for publicly available datasets
- A description of any restrictions on data availability
- For clinical datasets or third party data, please ensure that the statement adheres to our policy

All datasets generated and analysed during the current study are available in the Zenodo repository with the identifier doi.org/10.5281/zenodo.15001619

Research involving human participants, their data, or biological material

Policy information about studies with human participants or human data. See also policy information about sex, gender (identity/presentation), and sexual orientation and race, ethnicity and racism.

Reporting on sex and gender	We report on sex as a known risk factor for UTIs, in particular we highlight the link between sex and urethral length. We do not report on gender. Our model is applicable regardless of sex, but does contain a parameter that is determined by urethral length. We give values of this parameter for both males and females, but take as a default (for illustrative purposes) the value of the urethral length in females. However, in figure 4 we show results of varying urethral length, and discuss in detail how our results apply to both males and females. Where a sensitivity analysis was performed, the full physiological range of urethral lengths was taken, and results apply to both sexes. In figure 3 we show data processed from a publicly available dataset, showing the distribution of urine production rates in a population. We show this data disaggregated by sex. Where we make predictions of susceptibility to bacteriuria, and the effect of changing urine production rate on susceptibility to bacteriuria, our predictions are disaggregated by sex.
Reporting on race, ethnicity, or other socially relevant groupings	Our manuscript does not report on race, ethnicity or other socially relevant groupings.
Population characteristics	Our study does not have human research participants.
Recruitment	Our study does not have human research participants.
Ethics oversight	Our study does not have human research participants.

Note that full information on the approval of the study protocol must also be provided in the manuscript.

Field-specific reporting

Please select the one below that is the best fit for your research. If you are not sure, read the appropriate sections before making your selection.

☒ Life sciences ☐ Behavioural & social sciences ☐ Ecological, evolutionary & environmental sciences

For a reference copy of the document with all sections, see nature.com/documents/nr-reporting-summary-flat.pdf

Life sciences study design

All studies must disclose on these points even when the disclosure is negative.

Sample size	The majority of the study results are from a deterministic model, which has no sample size. For the sensitivity analysis (Table 2), the sample size (n=360448) was determined by checking for convergence of Sobol indices, as shown in Supplementary Figure 7b. For the population urine production rate data (Table S1), data was obtained from the CDC NCHS NHANES (https://www.cdc.gov/nchs/nhanes/), and the dataset constructed by combining multiple survey years as described at https://wwwn.cdc.gov/nchs/nhanes/tutorials/weighting.aspx, resulting in n=11011 females, and n=10202 males. For generating Figure 3b the data was constructed by a weighted resample, with n=100,000, with the sample size determined by checking agreement of the median/interquartile range of the resample with the weighted median/interquartile range of the original sample, as calculated in Stata.
Data exclusions	No data generated within this study was excluded. Data was generated by parameter ranges detailed within the manuscript, chosen to correspond with the physiological ranges found in the literature.
Replication	Our study is purely theoretical, with no experimental findings. All data can be reproduced from the code provided.
Randomization	There are no experimental groups within our study.
Blinding	There are no experimental groups within our study.

Reporting for specific materials, systems and methods

We require information from authors about some types of materials, experimental systems and methods used in many studies. Here, indicate whether each material, system or method listed is relevant to your study. If you are not sure if a list item applies to your research, read the appropriate section before selecting a response.

Materials & experimental systems

n/a	Involved in the study
☒	☐ Antibodies
☒	☐ Eukaryotic cell lines
☒	☐ Palaeontology and archaeology
☒	☐ Animals and other organisms
☒	☐ Clinical data
☒	☐ Dual use research of concern
☒	☐ Plants

Methods

n/a	Involved in the study
☒	☐ ChIP-seq
☒	☐ Flow cytometry
☒	☐ MRI-based neuroimaging

Plants

Seed stocks

Report on the source of all seed stocks or other plant material used. If applicable, state the seed stock centre and catalogue number. If plant specimens were collected from the field, describe the collection location, date and sampling procedures.

Novel plant genotypes

Describe the methods by which all novel plant genotypes were produced. This includes those generated by transgenic approaches, gene editing, chemical/radiation-based mutagenesis and hybridization. For transgenic lines, describe the transformation method, the number of independent lines analyzed and the generation upon which experiments were performed. For gene-edited lines, describe the editor used, the endogenous sequence targeted for editing, the targeting guide RNA sequence (if applicable) and how the editor was applied.

Authentication

Describe any authentication procedures for each seed stock used or novel genotype generated. Describe any experiments used to assess the effect of a mutation and, where applicable, how potential secondary effects (e.g. second site T-DNA insertions, mosiacism, off-target gene editing) were examined.
